# Effects of Acute Exercise on Cutaneous Thermal Sensation

**DOI:** 10.3390/ijerph17072491

**Published:** 2020-04-06

**Authors:** Samuel D. Thomas, Howard H. Carter, Helen Jones, Dick H.J. Thijssen, David A. Low

**Affiliations:** 1 Research Institute of Sports & Exercise Sciences, Liverpool John Moores University, Liverpool L3 3AF, UK; s.d.thomas@2016.ljmu.ac.uk (S.D.T.); howard.carter@uwa.edu.au (H.H.C.); h.jones1@ljmu.ac.uk (H.J.); d.thijssen@ljmu.ac.uk (D.H.J.T.); 2 Department of Physiology, Radboud University Nijmegen Medical Centre, 6525 GA Nijmegen, The Netherlands

**Keywords:** acute exercise, skin, sensory function, heat

## Abstract

The aim of this study was to assess the effect of exercise intensity on the thermal sensory function of active and inactive limbs. In a randomised and counterbalanced manner, 13 healthy young male participants (25 ± 6 years, 1.8 ± 0.1 m, 77 ± 6 kg) conducted: (1) 30-min low-intensity (50% heart rate maximum, HRmax; LOW) and (2) 30-min high-intensity (80% HRmax; HIGH) cycling exercises, and (3) 30 min of seated rest (CONTROL). Before, immediately after, and 1 h after, each intervention, thermal sensory functions of the non-dominant dorsal forearm and posterior calf were examined by increasing local skin temperature (1 °C/s) to assess perceptual heat sensitivity and pain thresholds. Relative to pre-exercise, forearm heat sensitivity thresholds were increased immediately and 1 hr after HIGH, but there were no changes after LOW exercise or during CONTROL (main effect of trial; *p* = 0.017). Relative to pre-exercise, calf heat sensitivity thresholds were not changed after LOW or HIGH exercise or during CONTROL (main effect of trial; *p* = 0.629). There were no changes in calf (main effect of trial; *p* = 0.528) or forearm (main effect of trial; *p* = 0.088) heat pain thresholds after exercise in either LOW or HIGH or CONTROL. These results suggest that cutaneous thermal sensitivity function of an inactive limb is only reduced after higher intensity exercise but is not changed in a previously active limb after exercise. Exercise does not affect heat pain sensitivity in either active or inactive limbs.

## 1. Introduction

The skin is a vital organ of regulation, helping maintain optimal cardiovascular, autonomic and sensory functions, among others, through its vast array of neural and morphological structures. Cutaneous thermal sensation plays a critical role in behavioural thermoregulation, which is the first line of defence against thermal disturbances [1]. Thermal sensation provides immediate feedback about the thermal state of the body and a level of thermal discomfort (the reciprocal of thermal comfort) is determined, and, if necessary, a set of desired actions, i.e., behaviours, are initiated to correct thermal imbalance/discomfort [2].

Exercise results in various responses to ensure optimal metabolic, cardiovascular and thermoregulatory function. For example, as heat production from active musculature increases, various neutrally mediated skin blood-flow and sweating reflexes occur in order to facilitate heat dissipation [3]. Despite these autonomic adjustments in order to serve cardiovascular and thermoregulatory functions, behavioural thermoregulation is still active during exercise and plays an important role in exercise intensity and local microclimate (i.e., seeking cooling and/or removing clothing) selection during exercise, ultimately to help limit thermal discomfort and avoid heat-related illness [4]. Furthermore, behavioural thermoregulation remains engaged after the cessation of exercise, which is particularly important due to the withdrawal of autonomic thermoeffectors [5] and the extended elevation of internal temperature and thermal discomfort post-exercise [6].

During and after exercise, sensory perceptions to a variety of different stimuli can be altered, including a reduction in pain sensation [7], or exercise-induced analgesia. The effect of exercise on thermal sensation is not entirely clear, however. Any changes in thermal sensation during or after exercise would have implications for behavioural thermoregulation and associated strategies. Minimal previous research has suggested a similar phenomenon to exercise-induced analgesia whereby arm and leg warmth sensation thresholds are increased, i.e., a higher skin temperature is required to generate the sensation of warmth during exercise [8], and the perceptual sensations of local cold (20 °C) and warmth (40 °C) stimuli are reduced, i.e., the same cold/heat applications are rated as less cold or hot, respectively, during low-intensity exercise [9,10,11]. With regards to thermal sensation post-exercise, recent work has shown that, compared with recovery after moderate-intensity exercise, during recovery from high-intensity exercise, thermal behaviour is withdrawn at a rate that is disproportionately high relative to the magnitude of changes in the afferent stimuli (e.g., core and skin temperatures) to continue behaving [12]. These findings are indicative of blunted thermal behaviour following high-intensity exercise and could be a result of attenuated perception of thermal afferent stimuli, e.g., thermal sensation, due to exercise-induced analgesia, which is more prevalent during/after high-intensities [7] and a carryover of reduced thermal sensation during exercise [8,9,10,11]. Thermal sensation after exercise, and any effect of the preceding exercise intensity, is relatively unknown, however [13]. Moreover, whether there is a regional variation in any changes in thermal sensation after exercise is also unknown. Regional variation in thermal sensation is evident under resting conditions [14,15] but whether thermal sensation is affected differently in previously active vs. inactive limbs is not known. The aim of this study was to therefore assess the effect of exercise intensity on the thermal sensory function of previously active and inactive limbs. The hypotheses are that (1) thermal sensory function would be impaired after high-intensity exercise but not after low-intensity exercise and (2) the thermal sensory function responses to exercise would not be different in the previously active leg and inactive forearm.

## 2. Materials and Methods

### 2.1. Participants

Participants (n = 13 males) who were recreationally active (as assessed by short IPAQ physical activity questionnaire, <4 sessions per week, $\dot{V}$O_2max_ 3.5 ± 0.5 L·min^−1^), healthy (as assessed by PARQ health screening form), young (age <45 years, mean = 25 ± 6 years), and non-smokers were recruited. Individuals with cardiovascular disease, local infections and limitations of physical activity, smokers, and persons taking medication were excluded. Participants were informed of the procedures prior to participation and provided written and verbal informed consent. This study was approved by the Liverpool John Moores University Research Ethics Committee in accordance with the Declaration of Helsinki (ref: 17SPS010). Height and weight measurements were collected and the assessment of $\dot{V}$O_2max_ was conducted at the first laboratory visit (mean height 1.8 ± 0.1 m, weight 77 ± 6 kg). The protocol for the $\dot{V}$O_2max_ involved an incremental cycling (Lode Corival CPET, Lode B. V., Groningen, The Netherlands) protocol to volitional exhaustion (30-watt increments every 2 min) while heart rate (Polar FT1 and T31, Polar, Kempele, Finland) and expired air (Jaeger Oxycon Pro, Wuerzburg, Germany) were continuously collected.

### 2.2. Experimental Design

Participants attended the laboratories on 3 occasions for 2 bouts of 30 min of exercise on a cycle ergometer (Lode Corival CPET, Lode B. V., Groningen, The Netherlands) at 50% (low-intensity exercise, LOW) or 80% (high-intensity exercise, HIGH) maximum heart rate (cadence of 0–90 rpm) or a control (CONTROL) no-exercise session when participants sat quietly for 30 min. Prior to (PRE), immediately following (IMM), and 1 h following the cessation of exercise (1HR), thermal sensory function of the non-dominant dorsal forearm and posterior calf were examined by increasing local skin temperature (1 °C/s) to assess heat sensitivity (detection of a change in skin temperature) and pain (detection of discomfort) thresholds. The order of the visits was randomised and counterbalanced, separated by 4–7 days, and were performed at the same time of day to minimise circadian variation [16]. Participants reported to the laboratories having fasted from food for 4 h, abstained from alcohol and caffeine for 16 h, and refrained from exercise 24 h prior to testing. Participants were advised to ingest 500 mL of water prior to testing to avoid dehydration. All testing visits took place in the same temperature-controlled room (23.3 ± 0.28 °C, 42 ± 7% relative humidity).

### 2.3. Thermal Sensory Function Assessment

Participants were positioned semi-recumbent for baseline stabilisation and thermal sensory function assessment. After instrumentation, resting baseline measurements were collected for 5 min. Non-dominant dorsal forearms and posterior calves were examined by increasing local skin temperature (1 °C/s) to assess heat sensitivity (detection of a warm sensation) and heat pain (detection of heat discomfort) thresholds (TSA II NeuroSensory Analyser, Medoc, Ramat Yishai, Israel) according to international consensus guidelines [17]. Five consecutive measurements were conducted for both warmth detection and heat pain detection thresholds. All thresholds were obtained with ramped stimuli (1 °C/s) that were terminated when the subject pressed a button. The contact area of the thermode was 7.84 cm^2^. The measurements for warmth and heat pain detection thresholds were not made in the same place during each set of 5 consecutive measurements to avoid any carryover effect from previous stimuli affecting a subsequent detection threshold.

### 2.4. Cardiovascular and Local Thermoregulatory Assessment

Intermittent systolic and diastolic blood pressure and heart rate were measured using an automated sphygmomanometer (Dinamap Procare 100, GE Medical Systems Ltd., Buckinghamshire, UK). Intra-exercise heart rate was continuously monitored using short-range telemetry (Polar FT1 and T31, Polar, Kempele, Finland). Local forearm and calf skin temperatures were recorded using thermocouples (Grant Instruments, Sheppreth, Cambridge, UK). Whole-body thermal discomfort (0–9 scale) [18] and ratings of perceived exertion (6–20 scale) [19] were assessed during the last 5 min of exercise.

### 2.5. Statistical Analysis

The median 3 results of the 5 trials at each stage were averaged for analyses of the warmth and heat pain thresholds. Separate two-factor linear mixed modelling with stage (2 levels: IMM vs. 1HR) and intensity (3 levels: LOW vs. HIGH vs. CONTROL) as factors were used to compare the changes in warmth and heat pain thresholds from baseline during the 3 trials at the forearm and calf. Haemodynamics and local skin temperature data were compared using linear mixed models, with main effects of stage and intensity. Baseline warmth and heat pain thresholds were compared using linear mixed modelling with intensity (3 levels: LOW vs. HIGH vs. CONTROL) as the single factor. Thermal discomfort and ratings of perceived exertion (RPE) data were compared between LOW and HIGH using paired *t*-tests. The normality of data distribution and homogeneity of variance were checked prior to statistical analyses, which were performed using SPSS (IBM SPSS Statistical Package 24). Statistical significance was set at *p* < 0.05 and data are expressed as mean ± 1 standard deviation (SD).

## 3. Results

### 3.1. Exercise Responses

By design, exercise work rate was significantly higher during HIGH compared to LOW (157 ± 28 vs. 82 ± 17 watts, *p* < 0.001). Exercise induced significant changes to all haemodynamic and local skin temperature variables whereas there were no changes during the CONTROL trial (Table 1). Exercise increased heart rate in an intensity-dependent manner (*p* < 0.001). Systolic and diastolic blood pressure increased during exercise (*p* < 0.001) with a higher systolic blood pressure during HIGH vs. LOW (*p* = 0.006) but no difference in diastolic blood pressure between HIGH and LOW (*p* = 0.633). Forearm skin temperature decreased during LOW exercise but was maintained during HIGH (*p* = 0.014). Calf skin temperature was higher during LOW and HIGH relative to CONTROL (*p* < 0.001) due to slight increases during and after exercise (*p* = 0.174). Ratings of perceived exertion were 10 ± 2 and 14 ± 2 for LOW and HIGH, respectively (*p* < 0.001). Thermal discomfort ratings were 5 ± 1 and 6 ± 1 for LOW and HIGH, respectively (*p* = 0.002).

### 3.2. Thermal Sensation Function

**Figure 1:**
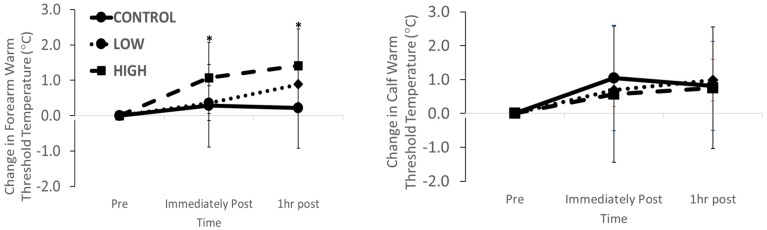
Changes in forearm and calf skin warm thermal sensitivity immediately and 1 hr after LOW and HIGH exercise and CONTROL * *p* < 0.05 vs. LOW and CONTROL.

Baseline forearm thermal sensation was not different between trials (CONTROL 35.0 ± 1.2 °C; LOW 34.3 ± 0.6 °C; HIGH 34.5 ± 0.5 °C; *p* = 0.073). There was a main effect of intensity for the change in forearm heat sensitivity threshold (*p* = 0.017) with an elevation immediately and 60 min after HIGH but no change after LOW or during CONTROL (Figure 1; stage*intensity effect; *p* = 0.210). Baseline calf thermal sensation was not different between trials (CONTROL 37.0 ± 1.3 °C; LOW 37.5 ± 1.7 °C; HIGH 37.1 ± 2.0 °C; *p* = 0.629). There was no main effect of stage (*p* = 0.840), intensity (*p* = 0.783) or stage*intensity interaction effect (*p* = 0.849) for the changes in calf heat sensitivity thresholds (Figure 1).

### 3.3. Thermal Pain Function

**Figure 2:**
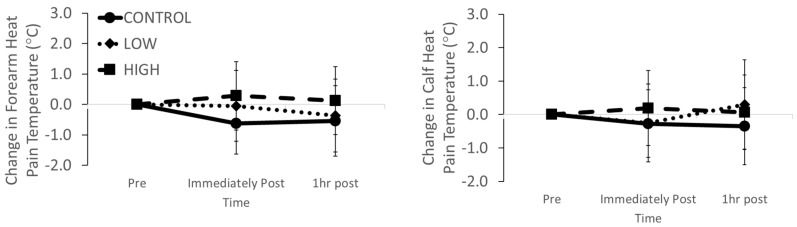
Changes in forearm and calf skin heat pain thresholds immediately and 1 hr after LOW and HIGH exercise and CONTROL.

The baseline forearm heat pain threshold was not different between trials (CONTROL 45.0 ± 3.0 °C; LOW 46.1 ± 2.2 °C; HIGH 45.7 ± 1.9 °C; *p* = 0.393). There was no main effect of stage (*p* = 0.551), intensity (*p* = 0.088) or stage*intensity interaction effect (*p* = 0.764) for the changes in forearm heat pain thresholds (Figure 2). Baseline calf thermal sensation was not different between trials (CONTROL 47.2 ± 1.6 °C; LOW 47.8 ± 1.8 °C; HIGH 47.4 ± 1.4 °C; *p* = 0.558). There was no main effect of stage (*p* = 0.683), intensity (*p* = 0.528) or stage*intensity interaction effect (*p* = 0.551) for the changes in calf heat sensitivity thresholds (Figure 2).

## 4. Discussion

The aim of this study was to assess the effect of exercise intensity on thermal sensory function of previously active and inactive limbs. Cutaneous thermal sensory function responses of the lower leg (calf) and forearm were assessed before, immediately and 1 hr after 30 min of low or high-intensity continuous cycling exercise. The main findings were (1) cutaneous thermal sensitivity of the forearm, e.g., a previously inactive limb, is reduced after high intensity exercise, consistent with previous findings during exercise [8]; (2) cutaneous thermal sensitivity of the lower leg, e.g., a previously active limb, is not changed after exercise in contrast to previous findings during exercise [8,9]; (3) exercise does not affect heat pain sensitivity in either previously active or inactive limbs, consistent with previous studies of heat pain sensitivity after exercise [13] but not other metrics of pain sensitivity after exercise [7].

Thermal sensation provides immediate feedback about the thermal state of the body and plays a critical role in behavioural thermoregulation, which is the first line of defence against exogenous thermal disturbances [1]. During thermal stress, despite intricate autonomic control of thermoeffectors, e.g., sweating and skin blood flow, that facilitate heat loss or gain in order to maintain internal temperature within safe limits, changes in levels of thermal discomfort [3] can initiate behaviour, e.g., finding shade or removing clothing, in order to also correct the thermal imbalance/discomfort [2]. The importance of effective thermal sensation and behavioural thermoregulation is particularly significant after exercise, when autonomic thermoeffectors are withdrawn despite the elevation of internal temperature post-exercise [6]. A collection of previous studies suggest that thermal sensation might be altered after exercise, however. During exercise, sensory perceptions of pain are reduced (exercise-induced analgesia) [7], and thermal sensation is impaired [8,9,10,11], which may carry over into the post-exercise period. Furthermore, recent work has suggested an attenuated perception of thermal afferent stimuli following high-intensity exercise due to a disproportionately high withdrawal of thermal behaviour [12]. In the present study, we showed that, using the warmth threshold detection limits during a local skin heating stimulus, cutaneous thermal sensitivity of the forearm was reduced immediately and 1 h after high intensity exercise, i.e., a higher skin temperature was required to generate the sensation of warmth. Such a phenomenon has been ascribed to exercise-induced activation of opioids [7,20]; proprioceptive and muscle afferents that inhibit central pain circuitry, which may involve modulation of descending inhibitory pathways [21]; the binding of released factors to pain and/or thermal receptors [22]; and/or distraction from pain or discomfort [23].

In the present study, cutaneous thermal sensitivity of the forearm was not different after the low-intensity exercise bout. Similar findings have also been demonstrated during low-intensity cycling (~30% $\dot{V}$O_2max_) [9]. It has been demonstrated that an exercise intensity of ≥75% $\dot{V}$O_2max_ is required to induce exercise-induced analgesia [7]. Given that the intensity of the LOW exercise in the current study was 50% of maximum heart rate, it is not surprising that thermal sensitivity in that condition was unchanged after exercise.

Interestingly, the thermal sensitivity of the calf was also not different after both low- and high-intensity exercise in the present study. Whether the thermal sensation of a limb that has been previously exercised, relative to an inactive limb, is affected is not clear. Several substances released by exercising muscles (e.g., potassium, hydrogen, prostaglandins) can activate or sensitize muscle nociceptors [22]. Whether any of these substances could also sensitize cutaneous nociceptors in active limbs and offset the reductions in thermal sensation observed in the non-active limbs is not known. An alternative explanation for the differing responses in the calf compared to the forearm in the present study could be regional variation in thermal sensitivity at rest and in response to exercise stimuli [14,15]. Previous research has demonstrated an exercise-induced reduction in calf cutaneous thermal sensitivity during exercise [9]. Differences between the findings of this previous work and the present study could be due to differences in the assessment method of thermal sensation; in the previous study, participants were asked to rate their thermal sensation after 10 s of 40 °C local heat application, i.e. via magnitude estimation, rather than indicate the sensation of warmth during an increasing local heat stimulus, i.e., via detection threshold. There were also differences in the timing (during vs. post-exercise) of thermal sensation assessments in both studies.

Exercise of low and high intensity did not affect heat pain sensitivity in either previously active or inactive limbs in the present study, consistent with previous research that reported a lack of change in heat pain thresholds after 30 min of moderate exercise in young participants [13], but not research that has demonstrated a reduction in other metrics of pain during and after exercise [7]. Exercise-induced analgesia is more consistently evident for methods that assess pain using tactile or electrical stimuli in comparison to equivocal findings for heat pain [7]. A lack of change in heat pain sensitivity (as well as calf heat sensitivity) in the present study may also be a result of the exercise intensity not being high enough and/or the exercise not being long enough.

The findings of this study have a range of implications, including for individuals exposed to heat after periods of exercise/physical activity. If thermal sensation and, subsequently, thermal behaviour are impaired after exercise, then the risk of heat-related illness may be elevated if an individual is subsequently exposed to heat stress, because they may not engage in optimal thermal behaviour to alleviate thermal discomfort. Furthermore, the findings could help inform clothing design that counteracts impaired thermal sensation of individuals exercising or working and exposed to heat stress.

There are some limitations to this study that are worthy of consideration. Given the regional variation in thermal sensation across the body [14,15], assessment of thermal sensation at additional sites, particularly on the torso or head, may have provided contrasting findings. Although local skin temperature at the sensation assessment sites was monitored, an index of core temperature was not recorded. It is highly likely that core temperature would have been elevated in the HIGH relative to the LOW trial and remained elevated for some time into the recovery period. Whether a higher exercise intensity would have further accentuated the changes in forearm thermal sensation and/or unmasked any changes in calf thermal sensation is also relevant. Finally, we chose to assess thermal sensation using the standard and commonly used method of thermal detection threshold. Alternative means of assessing thermal sensation, such as magnitude estimation via perceptual ratings of constant local heat stimuli, could have revealed complementary findings.

## 5. Conclusions

In conclusion, the findings of the present study indicate that cutaneous thermal sensitivity of an inactive limb is elevated only after higher intensity exercise, suggesting impaired sensory afferent function post-exercise, whereas thermal sensitivity of a previously active limb is not changed after exercise. Exercise does not affect heat pain sensitivity in either previously active or inactive limbs.

## Figures and Tables

**Table 1:** Cardiovascular and local skin temperature responses before (PRE), during (Ex), and immediately (IMM) and 1 hr (1HR) after 30 min of low and high intensity exercise and control rest. Data are mean ± 1 SD.

	Control				Low				High				*p* Values		
	PRE	Ex	IMM	1HR	PRE	Ex	IMM	1HR	PRE	Ex	IMM	1HR	Stage	Intensity	Stage * Intensity
Heart rate (beats·min)	64 ± 9	59 ± 10	59 ± 10	57 ± 8	61 ± 7	101 ± 4	65 ± 8	60 ± 9	58 ± 7	147 ± 7	86 ± 14	64 ± 11	<0.001	<0.001	<0.001
Skin temperature (°C)															
Forearm	32.3 ± 0.8	32.3 ± 1.0	32.4 ± 1.0	32.2 ± 1.0	32.3 ± 0.7	31.0 ± 0.9	31.3 ± 1.0	32.8 ± 1.1	32.2 ± 0.8	32.0 ± 0.8	31.9 ± 1.1	32.4 ± 1.1	0.006	0.050	0.014
Calf	31.0 ± 0.7	31.2 ± 1.0	31.2 ± 0.9	30.9 ± 0.9	31.1 ± 0.7	31.4 ± 1.5	31.8 ± 1.3	31.6 ± 1.0	31.5 ± 1.4	32.2 ± 0.9	32.0 ± 1.2	31.8 ± 0.9	0.174	<0.001	0.778
Blood pressure (mmHg)															
Systolic	122 ± 8	118 ± 9	119 ± 9	121 ± 8	118 ± 6	137 ± 11	124 ± 5	116 ± 9	121 ± 8	142 ± 17	124 ± 8	116 ± 8	<0.001	0.006	<0.001
Diastolic	67 ± 7	69 ± 7	68 ± 8	69 ± 8	65 ± 8	80 ± 12	67 ± 9	68 ± 9	66 ± 8	78 ± 11	67 ± 7	63 ± 6	<0.001	0.633	0.026
